# Effects of Endophyte Colonization of *Vicia faba* (Fabaceae) Plants on the Life–History of Leafminer Parasitoids *Phaedrotoma scabriventris* (Hymenoptera: Braconidae) and *Diglyphus isaea* (Hymenoptera: Eulophidae)

**DOI:** 10.1371/journal.pone.0109965

**Published:** 2014-10-22

**Authors:** Komivi S. Akutse, Komi K. M. Fiaboe, Johnnie Van den Berg, Sunday Ekesi, Nguya K. Maniania

**Affiliations:** 1 Plant Health Division, International Centre of Insect Physiology and Ecology (*icipe*), Nairobi, Kenya; 2 Unit of Environmental Sciences and Management, North-West University, Potchefstroom, South Africa; Federal University of Viçosa, Brazil

## Abstract

Effects of the fungal endophytes *Beauveria bassiana* (isolates ICIPE 279, G1LU3, S4SU1) and *Hypocrea lixii* (isolate F3ST1) on the life-history of *Phaedrotoma scabriventris* and *Diglyphus isaea*, parasitoids of the pea leafminer *Liriomyza huidobrensis*, were studied in the laboratory. Parasitoids were allowed to parasitize 2^nd^ and 3^rd^ instar *L. huidobrensis* larvae reared on endophytically-inoculated faba bean, *Vicia faba*. In the control, parasitoids were reared on non-inoculated host plants. Parasitism, pupation, adult emergence and survival were recorded. No significant difference was observed between the control and the endophyte-inoculated plants in terms of parasitism rates of *P. scabriventris* (*p = 0.68*) and *D. isaea* (*p = 0.45*) and adult' survival times (*p = 0.06*). The survival period of the F1 progeny of *P. scabriventris* was reduced (*p<0.0001*) in *B. bassiana* S4SU1 to 28 days as compared to more than 40 days for *B. bassiana* G1LU3, ICIPE 279 and *H. lixii* F3ST1. However, no significant difference (*p = 0.54*) was observed in the survival times of the F1 progeny of *D. isaea*. This study has demonstrated that together, endophytes and parasitoids have beneficial effects in *L. huidobrensis* population suppression.

## Introduction

Horticulture is the most important agricultural sector in Kenya. Arthropod pests do, however, present a major challenge to horticultural production. Among these pests, the invasive leafminer pests *Liriomyza huidobrensis* (Blanchard), *L. sativae* Blanchard and *L. trifolii* (Burgess) (all Diptera: Agromyzidae) pose the greatest challenge as they damage vegetable and ornamental crops [1], [2]. These pests also serve as vectors of plant pathogens [3], [4], [5], [6] and constitute quarantine pests in European markets [7], [8], [9].

The management of leafminers worldwide, and particularly in East Africa, has commonly relied on the frequent use of synthetic chemical insecticides [10], [11], [12], [13]. However, the indiscriminate and frequent use of these chemicals has resulted in insecticide resistance of flies [14], [2], pollution of the environment as well as elimination of their natural enemies [15], [2]. Chemical control is also not effective since flies usually escape insecticide applications due to their high mobility. Furthermore, *Liriomyza* larvae are inaccessible to many pesticides because they develop inside leaves and pupate in soil [16]. Horticultural producers are also under pressure to reduce pesticide use following the introduction of maximum residue levels (MRL) set up by the European Union on export produce. This has led to the search for more biorational management alternatives. Biological control using parasitoids, entomopathogenic fungi and fungal endophytes is being considered among the alternatives [17], [18]. Recently, Akutse *et al.* [18] demonstrated that fungal isolates of *Hypocrea lixii* (F3ST1) and of *Beauveria bassiana* (G1LU3, S4SU1 and ICIPE 279) could endophytically colonize *Vicia faba* and *Phaseolus vulgaris* plants and cause detrimental effects on life-history of *L. huidobrensis*. *Phaedrotoma scabriventris* Nixon (Braconidae: Opiinae) is an important leafminer parasitoid in Argentina, Brazil and Peru [19], [20], [16] and was introduced into Kenya for classical biological control of the leafminer flies (LMF). *Diglyphus isaea* Walker (Hymenoptera: Eulophidae) is one of the important indigenous parasitoids found in Kenya, Uganda and Tanzania [21] and is being used for the control of LMF in East Africa. Since both parasitoids and endophytic fungi may constitute key components of LMF management, understanding their interactions becomes crucial [22], [23]. Infection by fungal endophytes may affect the parasites of the insect herbivores feeding on host plants infected by the endophytes [24]. Effects may include reduction in fecundity, growth and survival of natural enemies, and even extend to changes in species richness and community structure of the parasitoid communities [25], [26], [27], [28]. However, the effect of the endophyte on the parasitoid can vary among the fungal isolates [28]. Most studies on multitrophic interactions, including arthropod communities and fungal endophytes, have been carried out on perennial ryegrass with fungal endophytes from grass [24], [28], [29], [30]. To our knowledge, there are no available reports on other systems. The objective of this study was, therefore, to investigate the multitrophic interactions between the host plant *Vicia faba*, the fungal endophytes, the pea leafminer *L. huidobrensis* and its ectoparasitoid *D. isaea*, and the endoparasitoid *P. scabriventris* system. Results show that the parasitoids' egg laying performance is not affected; thus, together with the examined endophytes, biological control of *Liriomyza* species seems promising; however, further analyses are required to validate this.

## Materials and Methods

### Ethics statement

The study was carried out at the International Centre of Insect Physiology and Ecology (*icipe*) laboratories in Kenya (S 03.35517° and E037.33861°) and not on private land. The plant (faba bean), endophytes and the insect pest (leafminer) involved in the study are not endangered or protected species. The fungal endophyte isolates were obtained from the *icipe*'s Arthropod Germplasm Centre and no permission was required since *icipe* operates under a Headquarters' agreement with the Kenyan Government. The parasitoid *Phaedrotoma scabriventris* was introduced into Kenya in 2008 following clearance by Peruvian Instituto Nacional de Recursos Naturales (INRENA) and by approval of the Kenya Standing Committee on Imports and Exports (KSTCIE). *Diglyphus isaea* is an indigenous parasitoid and no specific permission was required.

### Fungal cultures

*Beauveria bassiana* isolates G1LU3, S4SU1 and ICIPE 279, and *Hypocrea lixii* isolate F3ST1, previously reported pathogenic to *L. huidobrensis* [18] were used in this study. *Beauveria bassiana* isolates G1LU3, S4SU1 and *H. lixii* isolate F3ST1 were isolated from the aboveground parts of maize, sorghum and Napier grass [31] while *B. bassiana* isolate ICIPE 279 was isolated from an unidentified coleopteran larva. The isolates were cultured on potato dextrose agar (PDA) and maintained at 25±2°C in complete darkness. Conidia were harvested by scraping the surface of 2–3-week-old sporulating cultures with a sterile spatula. The harvested conidia were then mixed in 10 ml sterile distilled water containing 0.05% Triton X-100 and vortexed for 5 minutes to produce homogenous conidial suspensions. Conidial counts were done using a Neubauer hemacytometer chamber. The conidial suspension was adjusted to 1×10^8^ conidia ml^−1^.

Spore viability was determined before any bioassay using the technique described by Goettel & Inglis [32]. Conidia were deemed to have germinated when the length of the germ tube was approximately two times the diameter of the propagule/conidium. Four replicates were used for each isolate.

### Plant inoculation and endophyte colonization

Inoculation was done by soaking seeds of *V. faba* (a local Kenyan open pollinated bean variety) in conidial suspensions titrated at 10^8^ ml^−1^ for 2 hours. Prior to inoculation, seeds were surface-sterilized in 70% ethanol for 2 min followed by 1.5% sodium hypochlorite for 3 min after which seeds were rinsed three times with sterile distilled water. For the controls, sterilized seeds were soaked in sterile distilled water for 2 hours. The last rinse water was plated out to assess the effectiveness of the surface sterilization procedure [33]. Seeds were transferred into plastic pots (8 cm diameter×7.5 cm high) containing the planting substrate (mixture of manure and soil 1∶5). The substrate was sterilized in an autoclave for 2 hours at 121°C and allowed to cool for 72 hours prior to planting. Five seeds were planted per pot and maintained at room temperature (25±3°C and 60% RH). Pots were transferred to a screen house (2.8 m length×1.8 m width×2.2 m height) immediately after germination and maintained at 25±3°C for two weeks. Seedlings were thinned to three per pot after germination and were watered twice per day (morning and afternoon). No additional fertilizer was added to the substrate.

Endophytic colonization of the inoculated plants was confirmed using the technique described by Akutse et al. and Powell et al. [34], [18]. Plants were randomly selected and carefully removed from the pots two weeks after inoculation and the roots washed with tap water. Seedling leaves, stems and roots (ca. 30 cm in height) were cut into different sections (ca. 5 cm long). Five randomly selected leaf, stem and root sections from each plant were surface-sterilized as described above. The different plant parts were then aseptically cut into 1×1 cm pieces before placing the pieces 4 cm apart from each other, on PDA plates amended with a 0.05% solution of antibiotic (streptomycin sulfate salt) [35], [36], [37]. Plates were incubated at 25±1°C for 10 days, after which the presence of endophyte was determined. Prior to incubation of the different plant parts, the last rinse water was also plated out to assess the effectiveness of the surface sterilization procedure [33], [38]. The colonization of the different plant parts was recorded by counting the number of pieces that showed the inoculated fungal growth/mycelia according to Koch's postulates [39], [34]. Only the presence of the endophyte used for inoculation was scored. Mother slides were prepared from the mother plates for morphological identification. After colonization of the plant materials, new slides that were identical to the mother slides (for identification purposes) were prepared. The experiment was replicated three times over time.

### Insects

#### *Liriomyza huidobrensis*

*Liriomyza huidobrensis* was obtained from the Animal Rearing and Containment Unit (ARCU), International Centre of Insect Physiology and Ecology (*icipe*), Nairobi. The initial colony originated from adult leafminers collected from wild crucifers on the *icipe* campus (01°13.3′S 36°53.8′E, 1600 m.a.s.l.) and had been reared on *V. faba* for 8–10 generations prior to the experiments. Rearing colonies were maintained at 27±2°C with a photoperiod of 12L∶12D and relative humidity of approximately 40%. *Liriomyza huidobrensis* adults were fed on a 10% sucrose solution.

#### *Diglyphus isaea*

The ectoparasitoid *D. isaea* used in the experiments was also obtained from the ARCU, *icipe*. The colony originated from adult *D. isaea* collected from a leafminer-infested French bean, and from crucifer crops at Naivasha (S: 00.66731°; E: 036.38603°; 1906 m.a.s.l.), Kenya. *Diglyphus isaea* was reared on *L. huidobrensis*-infested *V. faba* in Plexiglas cages (50 cm×50 cm×45 cm) for 5–10 generations prior to experiments. Adult *D. isaea* were exposed to 2^nd^ and 3^rd^-instar larvae of *L. huidobrensis* and the colony was maintained at 27±2°C with a photoperiod of 12L∶12D and 40–50% RH. Adults were fed on a 10% honey solution.

#### *Phaedrotoma scabriventris*

The initial colony of *P. scabriventris* originated from Peru and was maintained at the quarantine facilities of *icipe* on 2^nd^ and 3^rd^ instar-infested *L. huidobrensis* for 8–10 generations prior to experiments. Adults were fed on a 10% honey solution.

### Effects of endophytically-colonized *Vicia faba* host plants on life-history of *Phaedrotoma scabriventris* and *Diglyphus isaea*

To obtain leafminer-infested plants with larvae of the appropriate size (2^nd^ and 3^rd^ instars), two-day-old mated adult flies (150 individuals at a sex ratio of 1∶2 male∶ female) were exposed for 48 hours to two-week-old endophytically-inoculated host plant seedlings in Plexiglas cages (50 cm×50 cm×45 cm). Each cage contained five potted plants and represented a treatment. Cages were maintained at 25±2°C, 50–70% RH and 12L∶12D photoperiod. All treatments were arranged in a randomized complete block design and the experiment replicated three times over time. After 48 hours post-exposure, an aspirator was used to remove flies from the cages to prevent excessive oviposition and feeding punctures damage by adult flies. Excessive punctures cause the destruction of a large number of cells and since males cannot make feeding punctures, they use the punctures made by females to feed on. In case food becomes scarce, females will continue to make punctures (feed and oviposit) on the same punctures, thereby affecting the already laid eggs or “excessive oviposition”. The inoculated-exposed plants were maintained until larvae reached the 2^nd^ and 3^rd^ instars (approximately 5–8 days post-exposure). The same procedure was used for the control but plants were not inoculated with fungal pathogens.

The endophytically-inoculated *V. faba* plants infested with 2^nd^ and 3^rd^ instar *L. huidobrensis* larvae were used for parasitoids exposure. Fifty *P. scabriventris* adults (in the sex ratio of 1∶2 males∶ females) and 50 *D. isaea* adults (in the sex ratio of 1∶2 males∶ females) were exposed separately to endophytically-inoculated infested plants for 48 hours, after which the exposed plants were removed and maintained to collect data on parasitoid pupal development.

Survival of exposed adult parasitoids was recorded by counting the number of live parasitoids on a daily basis inside the cages until all parasitoids died. Dead parasitoids were placed on Petri dishes lined with damp sterilized filter paper for any fungal growth on the surface of the cadaver (mycosis test). Pupae were harvested from leaves after 3–5 days post-exposure to parasitoids, counted and then incubated at 25±1°C until emergence. Adult emergence of both parasitoids and flies and sex ratio were determined and parasitism rates calculated. To determine adult survival, 20 adults of each parasitoid were selected from the above experiment (progenies) and their mortality/survival recorded daily until all parasitoids died. The parasitoids were maintained in a cage as described above. A 10% honey solution was provided as food and cages maintained at 25±1°C.

### Statistical analyses

Mortality, number of pupae, emergence and survival (for parent parasitoids and F1 progeny) data were analyzed using both analysis of variance (ANOVA) and survival analysis methods. The survival curves were generated using the Kaplan–Meir (K–M) method. The log-rank test was used to compare the effect of various isolates on survival of *P. scabriventris* and *D. isaea*.

The K–M estimator of the survivor function was:
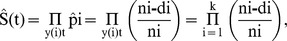
where y_(k)_≤t<y_(k+1)_, *n_i_* = the number in the risk set just before time *t*, *d_i_* = number died at time *y_(i)_*, *p_i_* = probability of surviving through the interval given being alive at the beginning of the interval, and *y*(*i*) denotes the *i*th distinct ordered censored or uncensored observation.

The number of pupae was log-transformed [Log_10_ (x+1)] before ANOVA analysis while the emergence and parasitism rates were square root-transformed [√(x+1)] before applying ANOVA analysis. Tukey HSD multiple comparisons of means was used to separate the means. The success rate (%) of parasitism was calculated as follows:
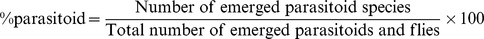
All the analyses were performed using R (2.13.1) statistical software [40] while relying heavily on the epicalc package [41].

## Results

### Effects of endophytically-colonized *Vicia faba* host plants on parasitism rates of *Diglyphus isaea* and *Phaedrotoma scabriventris*

The parasitism rate ranged between 15–33% with *D. isaea* and between 56–64% with *P. scabriventris* in endophytically-inoculated plants and was not significantly different with the control: *P. scabriventris* (*F = 0.59, df = 4, 9, p = 0.68*) and *D. isaea* (*F = 1.02, df = 4, 9, p = 0.45*) (Table 1).

**Table 1:** Percent parasitism of *Diglyphus isaea* and *Phaedrotoma scabriventris* following exposure to *Liriomyza huidobrensis* reared on endophytically-inoculated host plant.

	% Parasitism	
Fungal endophyte isolates	*Diglyphus isaea*	*Phaedrotoma scabriventris*
*Beauveria bassiana* S4SU1	16.3±9.3 a	55.7±1.7 a
*Beauveria bassiana* ICIPE279	15.5±8.9 a	62.2±1.9 a
*Beauveria bassiana* G1LU3	14.8±8.3 a	57.1±7.4 a
*Hypocrea lixii* F3ST1	33.4±12.8 a	63.6±5.6 a
Control	34.6±0.2 a	62.4±1.3 a

Means followed by the same letter within a column are not significantly different at 95% CI (p = 0.05).

### Effects of endophytically-colonized *Vicia faba* host plant on life history of *Diglyphus isaea* and *Phaedrotoma scabriventris* parasitizing *Liriomyza huidobrensis*

#### *Diglyphus isaea* adult survival

**Figure 1:**
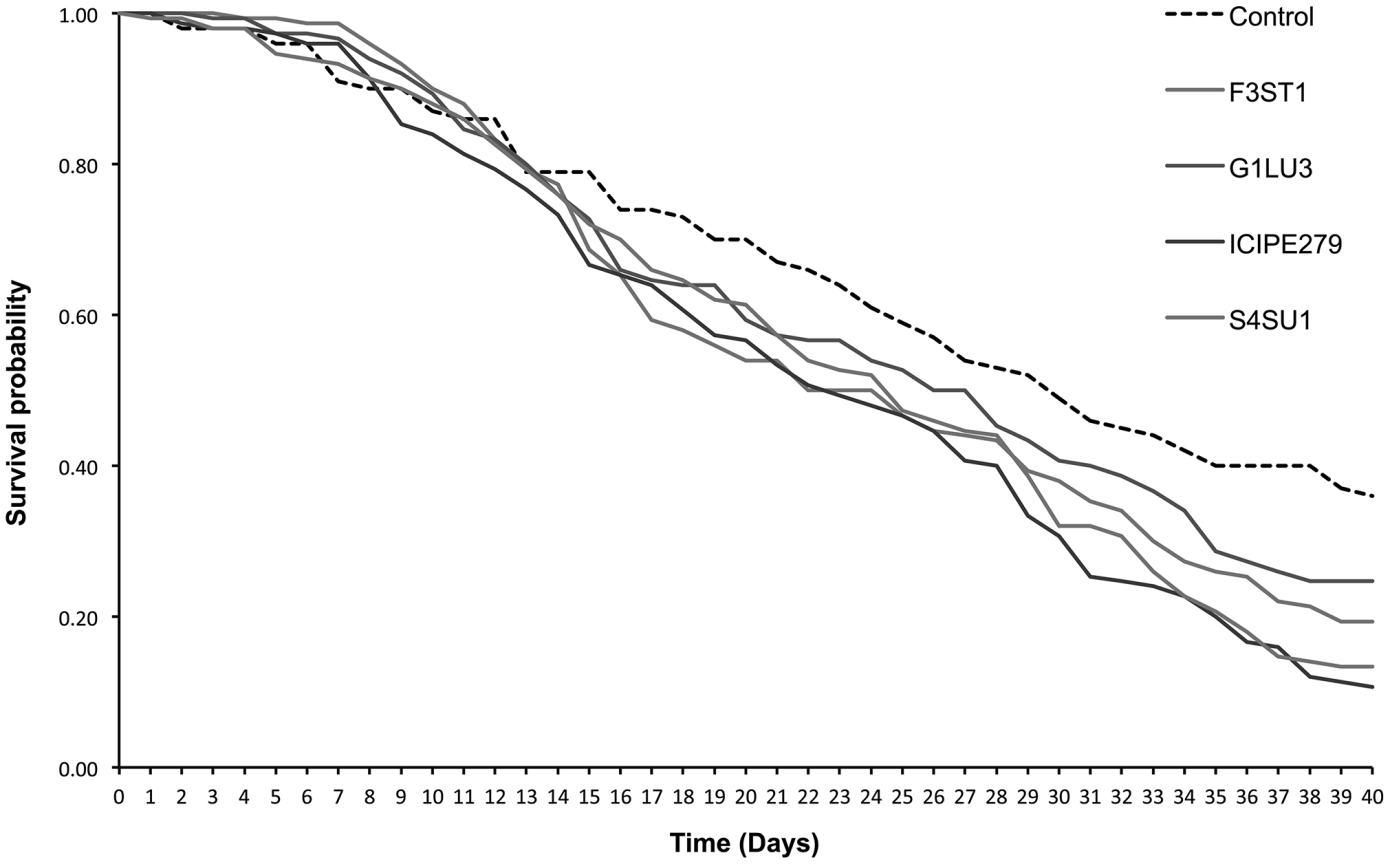
Survival curves for *Diglyphus isaea* adults parasitizing 2^nd^ and 3^rd^ instar *Liriomyza huidobrensis* larvae following exposure to *Vicia faba* plants endophytically-colonized by different fungal isolates of *Beauveria bassiana* (S4SU1, G1LU3 and ICIPE 279) and *Hypocrea lixii* F3ST1 after 40 days post-exposure.

Median survival time of *D. isaea* adults was 29.3 days in the control and varied between 21.2 and 24.5 days in fungal endophyte treatments, which were not significantly different (Table 2). The survival time curves showed no significant differences between the treatments including the control up to 14 days (*F = 2.3, df = 4, 555, p = 0.056*) but differed significantly among the treatments thereafter (*proximate log rank test = 19.48, df = 4, p<0.0001*) (Fig. 1). For example, at 29 days post-exposure, 52% *D. isaea* survived in the control while 43.3, 33.3, 38.7 and 39.3% in *B. bassiana* G1LU3, ICIPE 279, S4SU1 and *H. lixii*, respectively (Fig. 1). At 40 days post-exposure, the survival was 36.0% in the control and reduced to 10.7, 13.3, 24.7 and 19.3% in the *B. bassiana* ICIPE 279, S4SU1, G1LU3 and *H. lixii* F3ST1 treatments, respectively (Fig. 1).

**Table 2:** Mean survival time of *Diglyphus isaea* and *Phaedrotoma scabriventris* adult parents parasitizing *Liriomyza huidobrensis* following exposure to infested *Vicia faba* plants endophytically-colonized by the different endophyte fungal isolates.

	Mean survival time (Days) ± SE	
Fungal isolate species	*Diglyphus isaea*	*Phaedrotoma scabriventris*
*Beauveria bassiana* ICIPE 279	21.2±3.2 a	12.7±0.9 c
*Beauveria bassiana* G1LU3	24.3±3.1 a	18.5±1.0 b
*Beauveria bassiana* S4SU1	22.3±3.2 a	19.5±0.3 b
*Hypocrea lixii* F3ST1	24.5±4.8 a	21.5±1.2 ab
Control	29.3±2.7 a	26.0±1.0 a

Means followed by the same letter within a column are not significantly different at 95% CI (p = 0.05).

#### *Phaedrotoma scabriventris* adult survival

**Figure 2:**
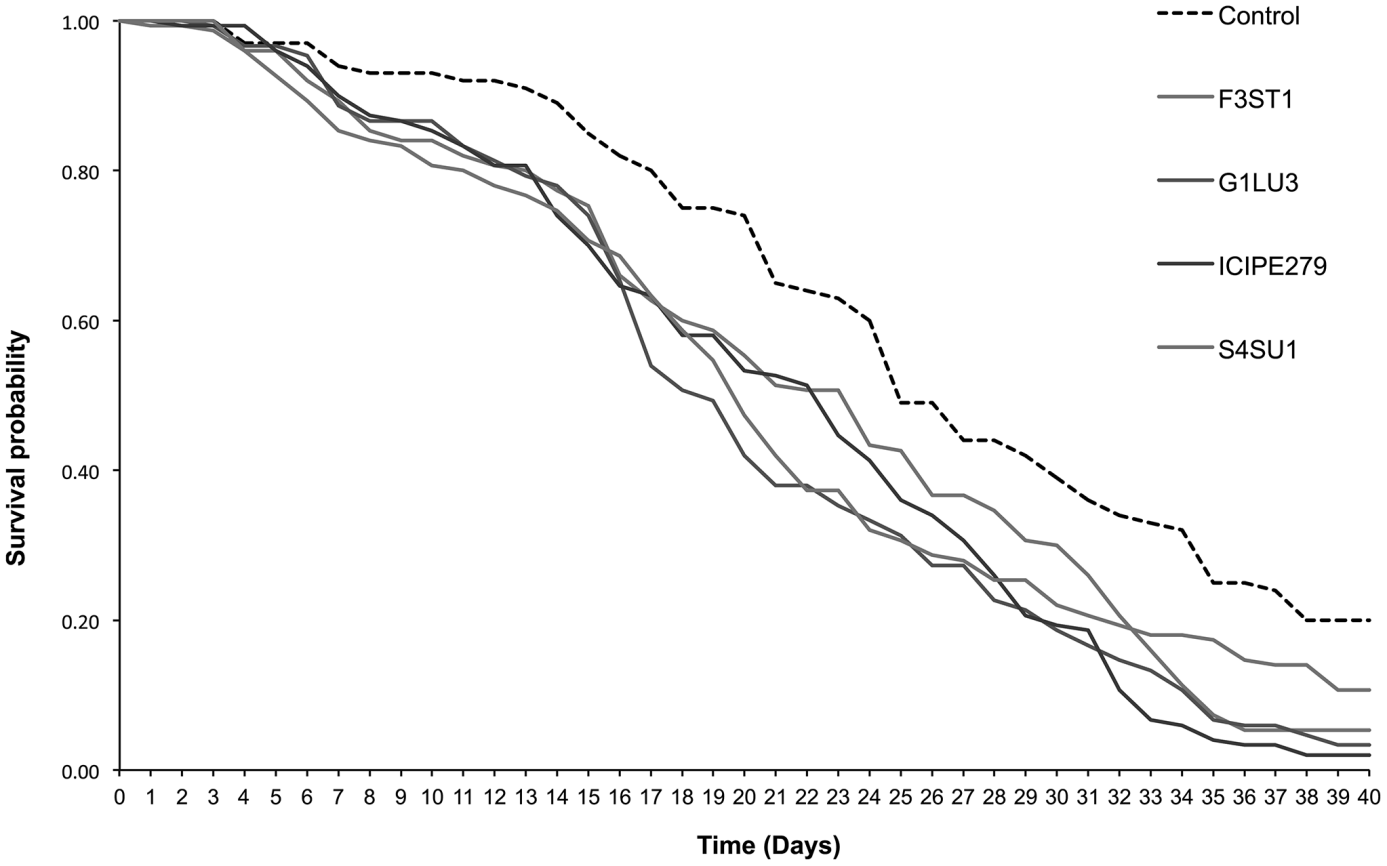
Survival curves for *Phaedrotoma scabriventris* adults parasitizing 2^nd^ and 3^rd^ instar *Liriomyza huidobrensis* larvae following exposure to *Vicia faba* plants endophytically-colonized by different fungal isolates of *Beauveria bassiana* (S4SU1, G1LU3 and ICIPE 279) and *Hypocrea lixii* F3ST1 after 40 days post-exposure.

Median survival times of *P. scabriventris* adults varied significantly between the treatments (*F = 23.64, df = 4, 9, p<0.0001*), with *B. bassiana* isolate ICIPE 279 having the shortest survival median time of 12.7 days and the control having the longest survival median time of 26.0 days (Table 2). The survival time curves also varied among treatments (*proximate log rank test = 26.32, df = 4, p<0.0001*) (Fig. 2). No significant differences in survival time curves were observed between the treatments during the first two weeks post-exposure but differed significantly thereafter. For example at 7 days post-exposure, survival time was higher than 90% for both *P. scabriventris* in the control and fungal endophyte treatments. At 14 days, more than 75% of survival was recorded in all the treatments including the control. However, at 21 days post-exposure, 65.0% survival was observed in the control while 38.0 and 42.0% in *B. bassiana* G1LU3 and S4SU1, respectively (Fig. 2). At 24 days post-exposure, the survival was reduced to 60.0 and 32.0% in the control and *B. bassiana* S4SU1, respectively. Further reduction was observed at 40 days post-exposure where 20.0% survival was recorded in the control and 10.7, 3.3, 2.0 and 5.3% in *B. bassiana* S4SU1, G1LU3, ICIPE 279 and *H. lixii* F3ST1, respectively (Fig. 2).

#### *Diglyphus isaea* pupation

**Figure 3:**
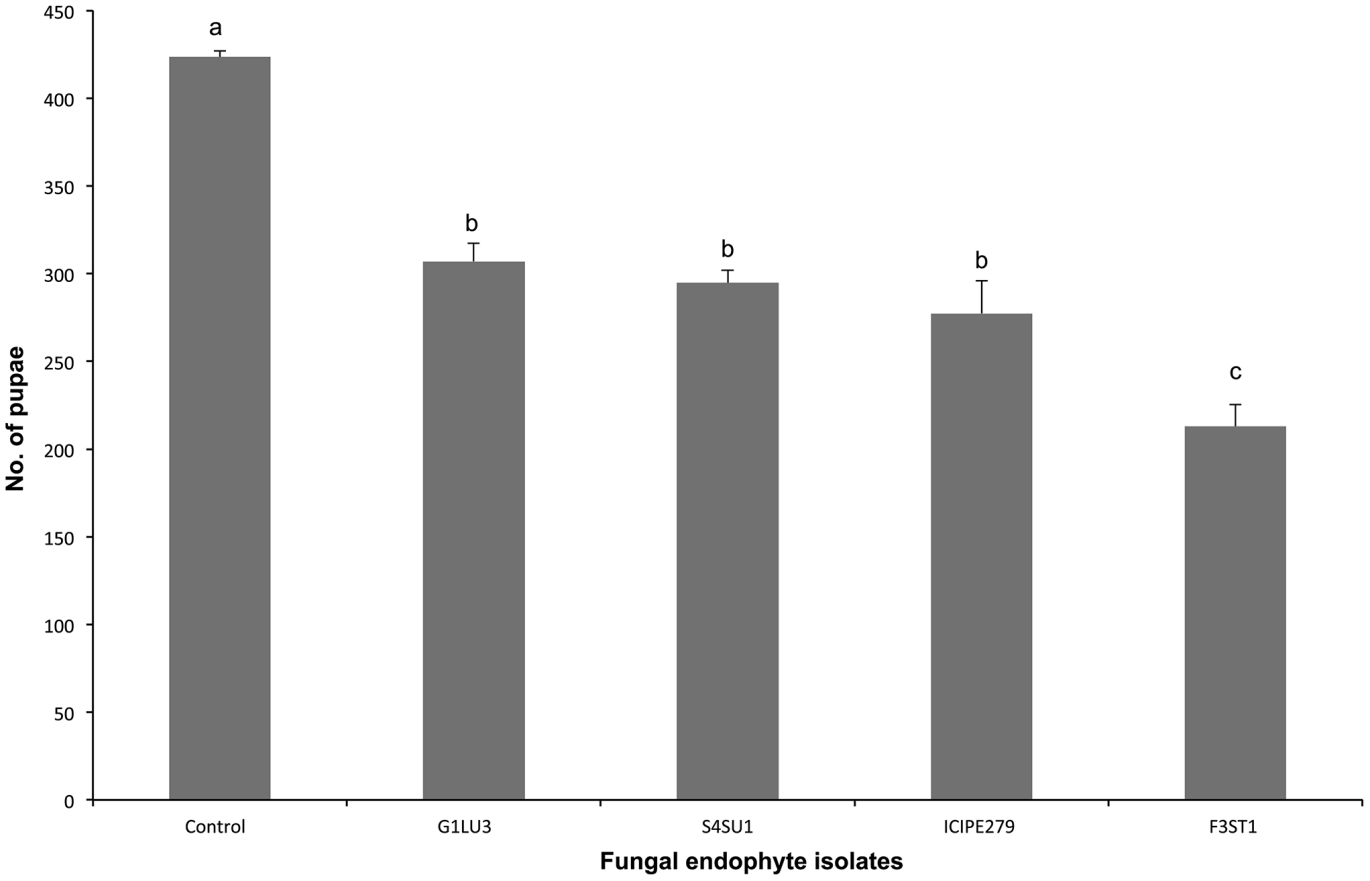
Effect of *Vicia faba* plants endophytically-colonized by different fungal isolates of *Beauveria bassiana* (S4SU1, G1LU3 and ICIPE 279) and *Hypocrea lixii* (F3ST1) and infested with 2^nd^ and 3^rd^ instar larvae of *Liriomyza huidobrensis* on the number of pupae produced after adult parents *Diglyphus isaea* exposure. Bars denote means ± one standard error at 95% CI (p = 0.05).

Fewer pupae of *D. isaea* were produced in endophytically-colonized plants (213.0±12.5–307.0±10.3) than in the control (423.5±3.5). There were, however, significant differences (*F = 30.40, df = 4, 9, p<0.0001*) among the fungal isolates, with *H. lixii* producing fewer pupae (Fig. 3).

#### *Phaedrotoma scabriventris* pupation

**Figure 4:**
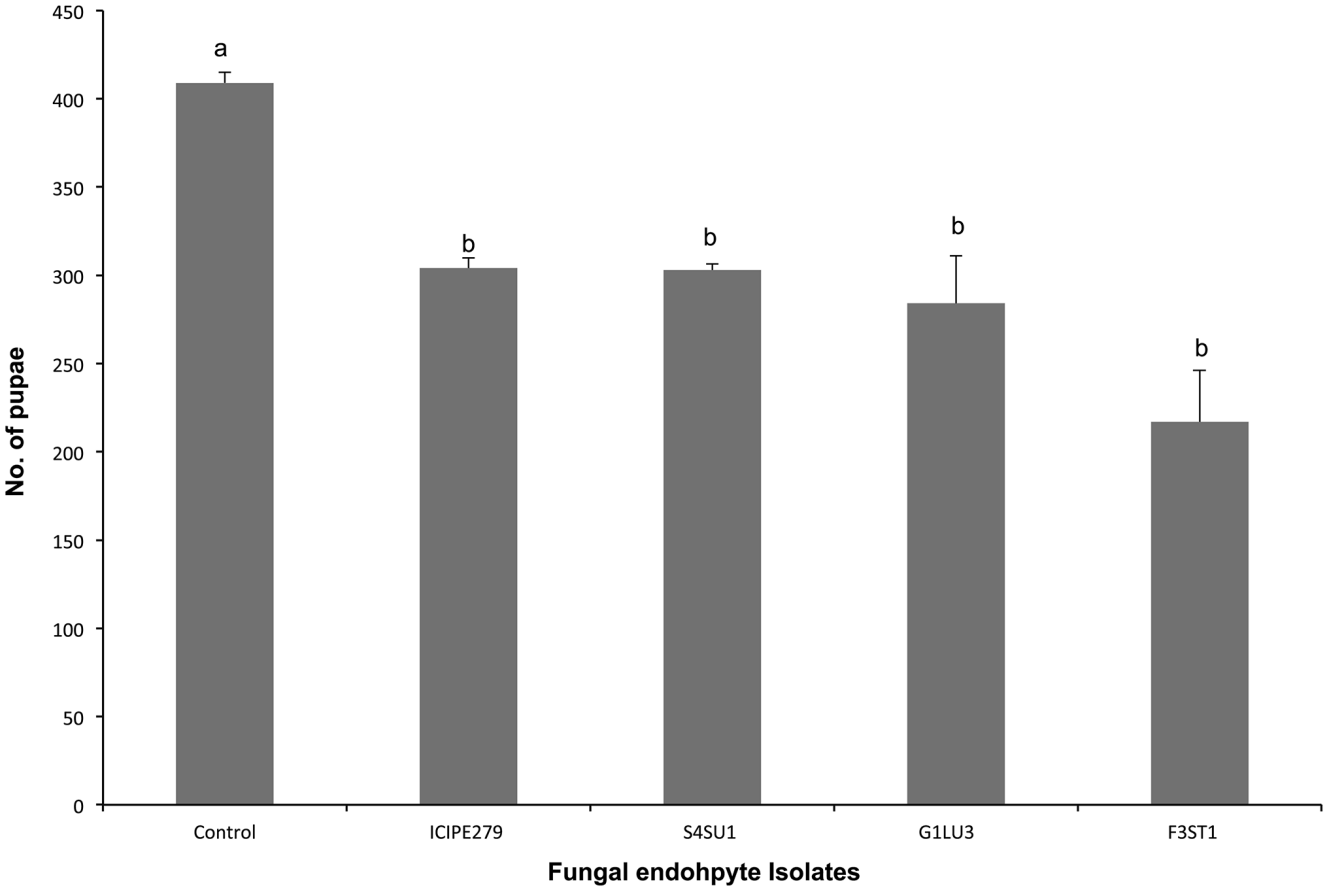
Effect of *Vicia faba* plants endophytically-colonized by different fungal isolates of *Beauveria bassiana* (S4SU1, G1LU3 and ICIPE 279) and *Hypocrea lixii* (F3ST1) and infested with 2^nd^ and 3^rd^ instar larvae of *Liriomyza huidobrensis* on the number of pupae produced after adult parents *Phaedrotoma scabriventris* exposure. Bars denote means ± one standard error at 95% CI (p = 0.05).

More pupae of *P. scabriventris* were produced in the control (409.0±6.0) than in endophytically-colonized plant treatments, which ranged between 217.0±9.0 and 304±6.0 (*F = 10. 29, df = 4, 9, p = 0.002*). However, no significant difference in the number of pupae was observed among fungal isolates (Fig. 4).

#### *Diglyphus isaea* adult emergence

**Figure 5:**
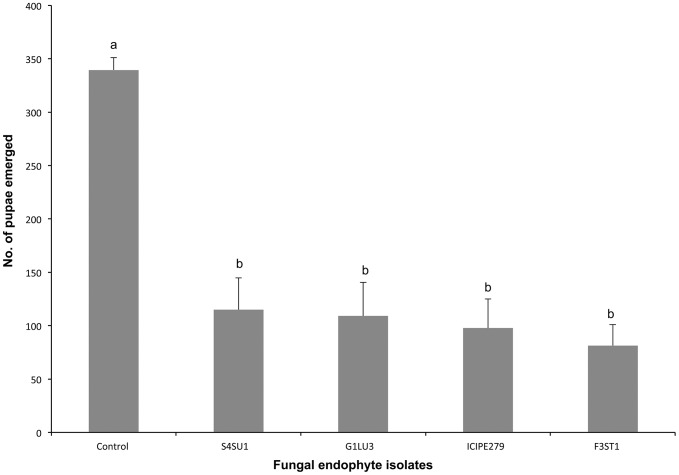
Effect of *Vicia faba* plants endophytically-colonized by different fungal isolates of *Beauveria bassiana* (S4SU1, G1LU3 and ICIPE 279) and *Hypocrea lixii* (F3ST1) on adult emergence of *Liriomyza huidobrensis* and *Diglyphus isaea*. Bars denote means ± one standard error at 95% CI (p = 0.05).

Higher numbers of flies emerged from pupae in the control plants (339.5±11.5) than from endophytically-colonized plants, which ranged between 81.3±9.7 and 115.0±9.8 (*F = 12.24, df = 4, 9, p<0.001*). There were no significant differences in adult emergence between fungal endophyte isolates (Fig. 5).

#### *Phaedrotoma scabriventris* adult emergence

**Figure 6:**
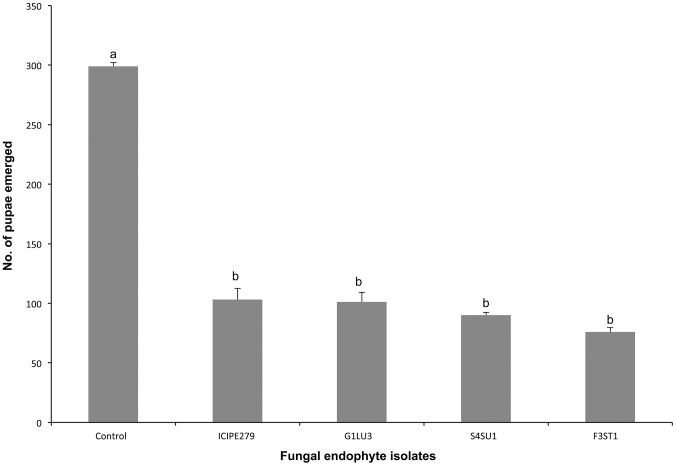
Effect of *Vicia faba* plants endophytically-colonized by different fungal isolates of *Beauveria bassiana* (S4SU1, G1LU3 and ICIPE279) and *Hypocrea lixii* (F3ST1) on adult emergence of *Liriomyza huidobrensis* and *Phaedrotoma scabriventris*. Bars denote means ± one standard error at 95% CI (p = 0.05).

As with *D. isaea*, higher numbers of flies emerged from control plants (299±3.1) than from endophytically-colonized plants, which varied between 76.0±3.1 and 103.0±9.5 (*F = 56.52, df = 4, 9, p<0.0001*). No significant differences were observed among the four fungal endophyte isolates (Fig. 6).

#### Sex ratio

There were no significant differences in sex ratio between males and females among fungal endophyte isolate and control treatments in *D. isaea* (*F = 0.75, df = 4, 9, p = 0.58*) and in *P. scabriventris* (*F = 0.98, df = 4, 9, p = 0.47*).

No mycosis was observed among all the 1500 cadavers of *D. isaea* and *P. scabriventris* exposed to endophytically-colonized and infested *V. faba* plants.

### Effects of fungal endophyte isolates on survival of *Diglyphus isaea* and *Phaedrotoma scabriventris* progenies

#### *Diglyphus isaea* progeny survival

**Figure 7:**
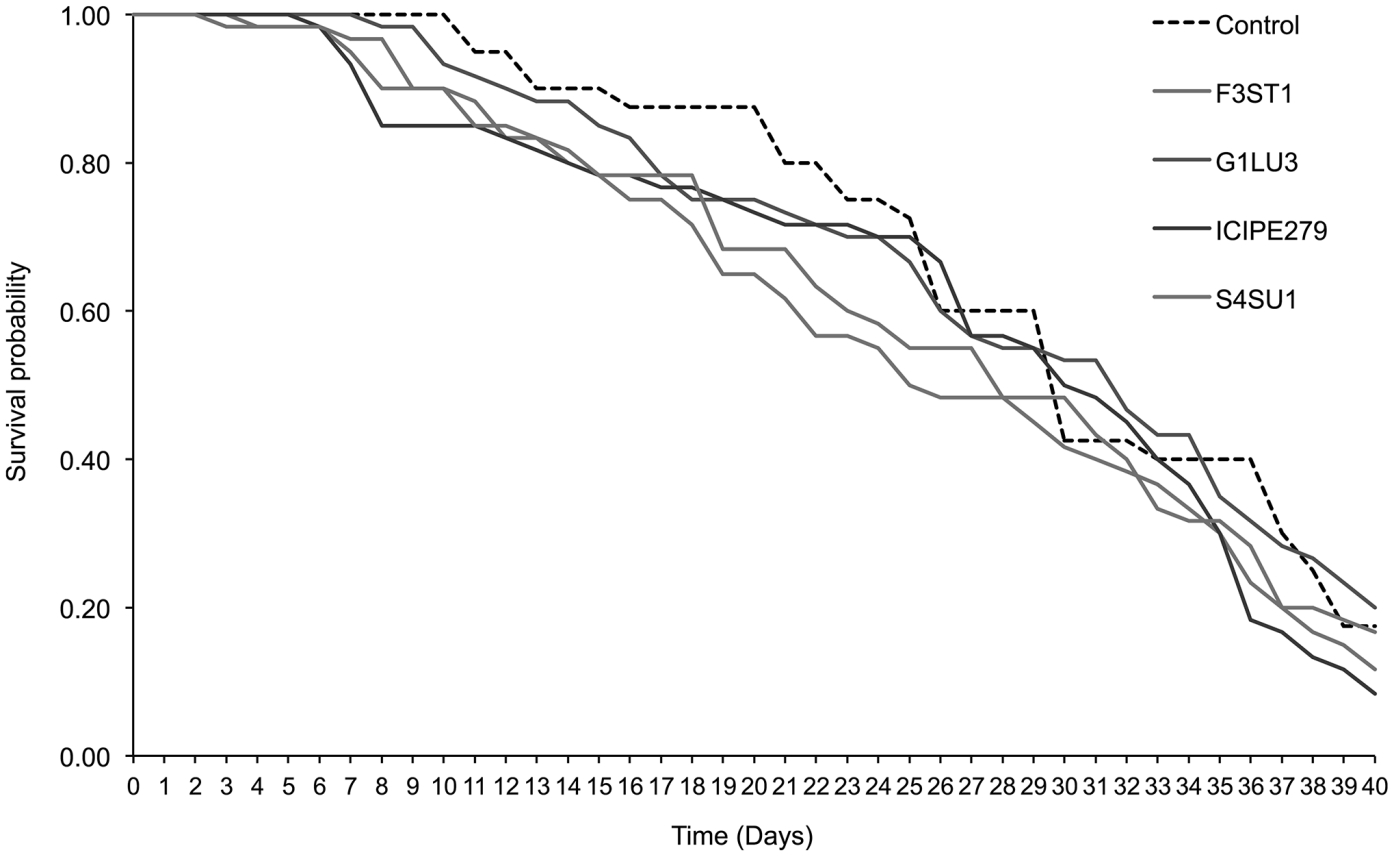
Progeny survival curves of *Diglyphus isaea* emerging from *Vicia faba* plants endophytically-colonized by different fungal isolates of *Beauveria bassiana* (S4SU1, G1LU3 and ICIPE279) and *Hypocrea lixii* (F3ST1) and infested with 2^nd^ and 3^rd^ instar larvae of *Liriomyza huidobrensis*.

The median survival times of the F1 *D. isaea* progenies (whose parents were previously exposed to endophytically-inoculated and untreated plants) varied between 27.0 and 30.3 days and was not significantly different among the treatments (*F = 0.34, df = 4, 9, p = 0.84*) (Table 3). Similarly, adult survival curves of the progeny did not differ significantly among the treatments (*proximate log rank test = 3.127, df = 4, p = 0.5367*) (Fig. 7).

**Table 3:** Mean survival time of *Diglyphus isaea* and *Phaedrotoma scabriventris* F1 progeny whose parents were exposed to *Liriomyza huidobrensis*-infested *Vicia faba* plants colonized by the different endophyte fungal isolates.

	Mean survival time (Days) ± SE	
Fungal isolate species	*Diglyphus isaea*	*Phaedrotoma scabriventris*
*Beauveria bassiana* ICIPE 279	28.7±2.8 a	20.5±3.9 a
*Beauveria bassiana* G1LU3	29.5±1.7 a	20.3±3.1 a
*Beauveria bassiana* S4SU1	27.5±2.0 a	14.5±3.2 a
*Hypocrea lixii* F3ST1	27.0±2.9 a	30.3±8.7 a
Control	30.3±2.8 a	28.0±6.0 a

Means followed by the same letter within a column are not significantly different at 95% CI (p = 0.05).

#### *Phaedrotoma scabriventris* progeny survival

**Figure 8:**
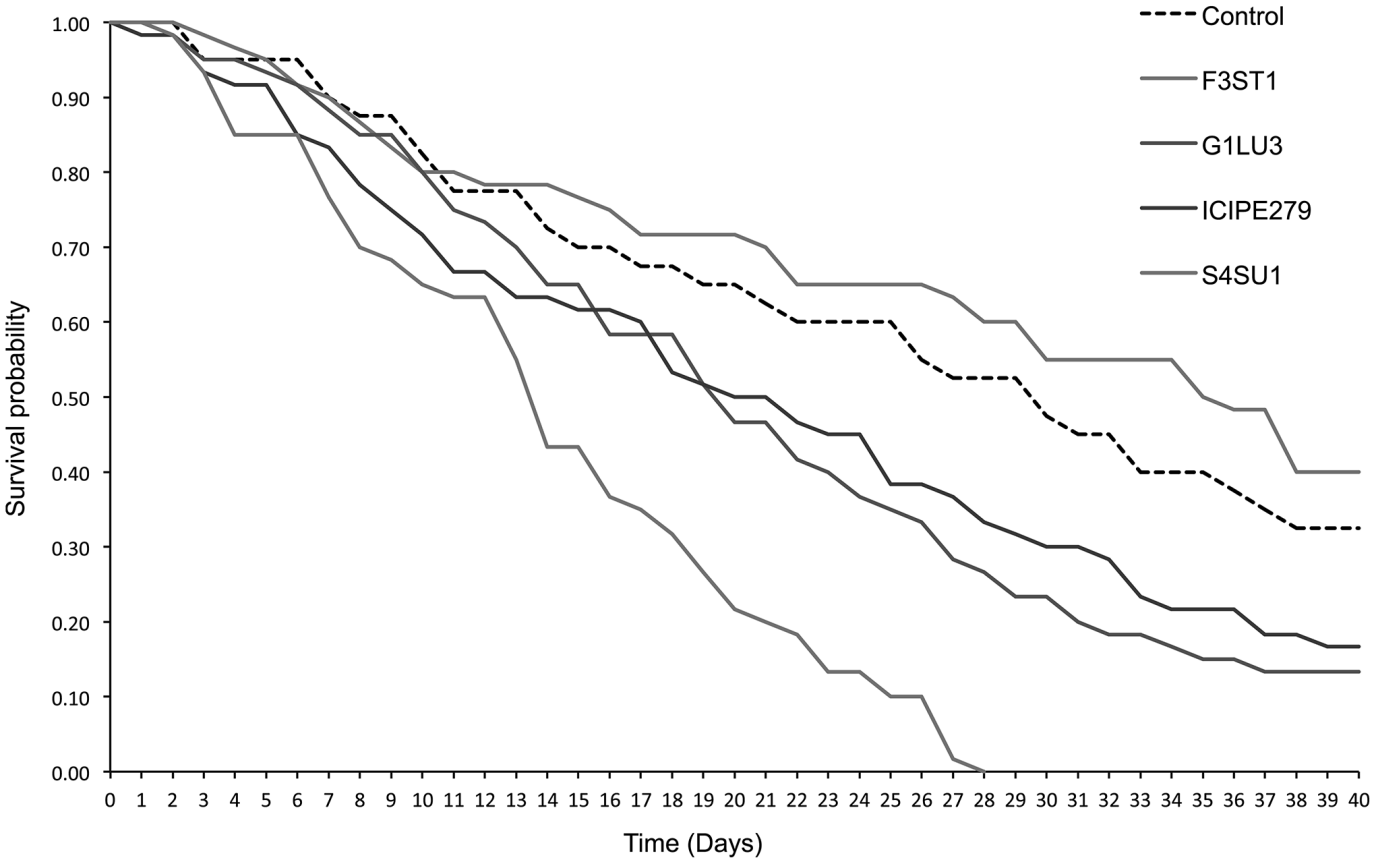
Progeny survival curves of *Phaedrotoma scabriventris* emerging from *Vicia faba* plants endophytically-colonized by different fungal isolates of *Beauveria bassiana* (S4SU1, G1LU3 and ICIPE279) and *Hypocrea lixii* (F3ST1) and infested with 2^nd^ and 3^rd^ instar larvae of *Liriomyza huidobrensis*.

The median survival times of the F1 *P. scabriventris* progenies were not significantly different among the treatments (*F = 1.42, df = 4, 9, p* = 0.304) (Table 3). However, the survival curves of the progeny differed significantly among the treatments (*proximate log rank test = 56.473, df = 4, p<0.0001*) (Fig. 8). At 7 days post-emergence, survival was approximately 90% in all the treatments, except in *B. bassiana* S4SU1 where it was 76.7%. At day 28, no survival (0%) was observed in *B. bassiana* S4SU1 while it was 60, 52.5, 33.3 and 26.7% in *H. lixii* F3ST1, control, *B. bassiana* ICIPE 279 and G1LU3, respectively (Fig. 8). At day 40, the lowest survival of progeny was recorded in *B. bassiana* ICIPE G1LU3, followed by *B. bassiana* ICIPE 279 (Fig. 8).

## Discussion

Results of the present study indicate that parasitism rates of *L. huidobrensis* by both *P. scabriventris* and *D. isaea* were not affected by endophytic colonization of *V. faba* plants by fungal isolates on which host insect was reared. Similar results were reported by Barker & Addison [42] on *Microctonus hyperodae* Loan (Hymenoptera: Braconidae), a parasitoid of *Listronotus bonariensis* (Kuschel) (Coleoptera: Curculionidae) on ryegrass infected with fungal endophyte *Acremonium lolii* Latch, Christensen & Samuels (Ascomycetes: Clavicipitaceae), and Härri *et al.* [43] on *Aphidius ervi* Haliday (Hymenoptera: Aphididae), parasitoid of the aphid *Metopolophium festucae* (Stroyan) (Hemiptera: Aphididae) on *Lolium perenne* L. (Cyperales: Poaceae).

The lowest number of pupae produced by *D. isaea* was recorded on plants endophytically-colonized by *H. lixii* F3ST1. However, this result does not compromise the performance (parasitism rates) of the parasitoid but rather confirms the results reported by Akutse *et al.* [18] where *H. lixii* F3ST1 reduced the number of pupae by causing high larval mortality as well as adult emergence of *L. huidobrensis*. This may be explained by the fact that few larvae reached pupation stage in the endophyte treatments as a result of larval parasitization. On the other hand, the number of pupae produced by *P. scabriventris* did not vary significantly among the endophytically-colonized plants. The lowest number of pupae recorded in *D. isaea* compared to *P scabriventris* in the endophytically-colonized *V. faba* plants may be due to the feeding and stinging activity of the ectoparasitoid *D. isaea* compared to the endoparasitoid *P. scabriventris*. Liu *et al.* [44] reported that *D. isaea* caused the death of host larvae not only by reproductive host-killing through parasitization, but also by non-reproductive host-killing by feeding and/or stinging without oviposition. Mafi & Ohbayashi [45] also reported that feeding and stinging without oviposition by *Sympiesis striatipes* Ashmead (Hymenoptera: Eulophidae), an ectoparasitoid of the citrus leafminer *Phyllocnistis citrella* Stainton (Lepidoptera: Gracillariidae), killed 44.7±4.2% of the host larvae per female parasitoid. Higher number of flies emerged from pupae in the control plants as compared to endophytically-inoculated hosts, which demonstrates the combined effects of the endophytes and the parasitoids in *L. huidobrensis* suppression. Chabi-Olaye *et al.* [46] had earlier reported on high parasitism rates of *Liriomyza* species by *P. scabriventris* in the laboratory. On the other hand, Akutse *et al.* [18] reported the negative effects of fungal endophytes on *L. huidobrensis* life-history parameters. Thus, endophytes and parasitoids may act complementarily to suppress *L. huidobrensis* population.

No negative effects of endophytically-inoculated plants were observed on survival of the parent parasitoids as well as their respective progenies. Bultman *et al.* [27] also found no effects of endophyte-infected tall fescue grass, *Festuca arundinacea* (Schreb.) Darbysh. (Cyperales: Poaceae), on survival of the parasitoids, *Euplectrus comstockii* Howard and *Euplectrus plathypenae* Howard (Hymenoptera: Eulophidae), of the fall armyworm, *Spodoptera frugiperda* (J.E. Smith) (Lepidoptera: Noctuidae). In contrast, Rodstrom *et al.* [47] observed higher survival for *E. comstockii* reared from hosts fed on plants free of fungal infection as compared to those reared from hosts fed on plants infected with two fungal isolates (AR542 and CS). Although no data were generated on the development of the parasitoids, results seem to indicate that development was not affected as reported by Bixby-Brosi & Potter [26] on *Linnaemya comta* (Fallen) (Diptera: Tachinidae) in its host *Agrotis ipsilon* Hufnagel (Lepidoptera: Noctuidae) fed on perennial ryegrass containing an alkaloid-producing fungal endophyte.

Since more than 75% of the two parasitoid populations survived 14 days after exposure, they would have the time to lay enough eggs and parasitize *L. huidobrensis* larvae during their life span. At 15°C development time from egg to adult is 26–27 days, while at 25°C this is shortened to 10–11 days [48], [49] which was the temperature used in our experiments. However, since *D. isaea* can survive more than 40 days post-exposure on endophytically-inoculated host plant, it will continue to lay enough eggs and subsequently generate many offsprings. Similarly, *P. scabriventris* development time is between 12–15 days at 25°C [50] and can survive for more than 40 days post-exposure to larval feeding on endophytically-inoculated host plant (Fig. 2). It can therefore produce many generations and parasitize many larvae before the end of its life span.

Since solitary parasitoids like *P. scabriventris* and *D. isaea* often kill hosts earlier in their larval development than gregarious ones whose offspring need more resources for development [51], they may escape the secondary chemicals (metabolites which may be produced by endophytically-colonized host plants) on which the larvae of *L. huidobrensis* may feed on before being parasitized [52], [53]. According to Bixby-Brosi & Potter [26], *Copidosoma bakeri*, a polyembryonic wasp that develops from egg to adult within the host, would suffer greater negative fitness effects than would *Linnaemya comta* (Fallén) (Diptera: Tachinidae), a solitary, rapidly developing parasitoid, when their common host feeds on alkaloid-containing endophytic grass. The same authors reported that proportionately fewer parasitized cutworms yielded *C. bakeri* broods when the caterpillars consumed endophyte-inoculated grass. The tachinid, in contrast, did not appear to be affected by the presence of endophyte infection within its host plant. As solitary parasitoids [26], *P. scabriventris* and *D. isaea* often spend less time in *L. huidobrensis* host larvae than polyembrionic parasitoids. Lampert & Bowers [54] also reported a similar result on the generalist *Trichoplusia ni* (Hübner) (Lepidoptera: Noctuidae) as a host for the polyembryonic parasitoid *Copidosoma floridanum* Ashmead (Hymenoptera: Encyrtidae).

Endophytically-inoculated plants can suppress insect pests [55], [56], [18] and could play an important role as a component of integrated pest management (IPM) of *L. huidobrensis*. However, the beneficial value can be compromised if the option is not compatible with other IPM components such as parasitoids. Our results showed that *B. bassiana* S4SU1 reduced the progeny survival time of *P. scabriventris* in inoculated *V. faba* plant as compared to the other fungal endophytes. However, although *B. bassiana* S4SU1 reduced the survival times of the exposed parasitoid parents, it had no effects on the progeny survival times.

## Conclusion

Endophyitc colonization of *V. faba* plants by fungal isolates did not have negative effects on the parasitism rates of *L. huidobrensis* by both *P. scabriventris* and *D. isaea*. However, higher number of flies emerged from pupae in the control plants compared to endophytically-treated hosts as a result of the effects of endophytes and parasitoids on *L. huidobrensis* population. Additionally, no negative effects were observed on the survival of the exposed parent parasitoids as well as their respective progenies. This study highlights the multitritrophic interactions between endophytic fungi, parasitoids, and host plant, which may vary according to the parasitoid species. Since the survival and parasitism rates (parasitoids performance) of both parasitoids were not affected, they may lay enough eggs during their life span to reduce the host pests' population. These endophytes can then be used in combination with the two parasitoids to control *Liriomyza* species. However, further study will be required to validate these results.
